# Associations between Urinary and Dietary Selenium and Blood Metabolic Parameters in a Healthy Northern Italy Population

**DOI:** 10.3390/antiox10081193

**Published:** 2021-07-26

**Authors:** Teresa Urbano, Tommaso Filippini, Daniela Lasagni, Tiziana De Luca, Sabrina Sucato, Elisa Polledri, Francesco Bruzziches, Marcella Malavolti, Claudia Baraldi, Annalisa Santachiara, Thelma A. Pertinhez, Roberto Baricchi, Silvia Fustinoni, Marco Vinceti

**Affiliations:** 1 CREAGEN—Environmental, Genetic and Nutritional Epidemiology Research Center, Department of Biomedical, Metabolic and Neural Sciences, University of Modena and Reggio Emilia, 41125 Modena, Italy; teresa.urbano@unimore.it (T.U.); tommaso.filippini@unimore.it (T.F.); francescobruzziches@hotmail.it (F.B.); marcella.malavolti@unimore.it (M.M.); 2 Transfusion Medicine Unit, Azienda USL-IRCCS of Reggio Emilia, 42123 Reggio Emilia, Italy; dani.lasagni@gmail.com (D.L.); Tiziana.DeLuca@ausl.re.it (T.D.L.); thelma.deaguiarpertinhez@unipr.it (T.A.P.); roberto.baricchi@ausl.re.it (R.B.); 3 Department of Clinical Sciences and Community Health, University of Milan, 20122 Milan, Italy; sabrina.sucato@unimi.it (S.S.); elisa.polledri@unimi.it (E.P.); silvia.fustinoni@unimi.it (S.F.); 4 Department of Biomedical, Metabolic and Neural Sciences, University of Modena and Reggio Emilia, 41125 Modena, Italy; claudia.baraldi@unimore.it; 5 AVIS Provinciale, 42013 Reggio Emilia, Italy; annalisa.santachiara@avisre.it; 6 Department of Medicine and Surgery, University of Parma, 43125 Parma, Italy; 7 IRCCS Ca’ Granda Foundation Maggiore Policlinico Hospital, 20122 Milan, Italy; 8 Department of Epidemiology, Boston University School of Public Health, Boston, MA 02118, USA

**Keywords:** dietary selenium, urinary selenium, biomarkers of exposure, glucose levels, lipid blood profile

## Abstract

Selenium is both an essential nutrient and a highly toxic element, depending on its dose and chemical forms. We aimed to quantify urinary selenium excretion and dietary selenium intake in 137 healthy non-smoking blood donors living in the northern Italian province of Reggio Emilia. We assessed selenium status by determining urinary selenium levels (mean 26.77 µg/L), and by estimating dietary selenium intake (mean 84.09 µg/day) using a validated semi-quantitative food frequency questionnaire. Fasting blood levels of glucose, lipids and thyroid-stimulating hormone were measured using automatized laboratory procedures. Dietary and urinary selenium were correlated (beta coefficient (β) = 0.19). Despite this, the association of the two indicators with health endpoints tended to diverge. Using linear regression analysis adjusted for age, sex, body mass index, cotinine levels and alcohol intake, we observed a positive association between urinary selenium and blood triglyceride (β *=* 0.14), LDL-cholesterol (β = 0.07) and glucose levels (β = 0.08), and an inverse one with HDL-cholesterol (β = −0.12). Concerning dietary selenium, a slightly positive association could be found with glycemic levels only (β = 0.02), while a negative one emerged for other endpoints. The two selenium indicators showed conflicting and statistically highly imprecise associations with circulating TSH levels. Our findings suggest that higher selenium exposure is adversely associated with blood glucose levels and lipid profile. This is the case even at selenium exposures not exceeding tolerable upper intake levels according to current guidelines.

## 1. Introduction

Over the last decades, a number of studies have tried to elucidate the controversial and intriguing role of the trace element selenium in human health. Selenium has a large spectrum of both nutritional and toxicological properties in humans [1], animals [2,3] and plants [4], with a still uncertain balance between the two. In fact, the safe range of intake is not well defined and is still debated. Different choices have been made by different agencies based on very different rationales, while there is increasing awareness of the potential for low-dose selenium toxicity [5,6].

Several diseases and conditions have been linked to selenium deficiency and excess [6]. Over time, emphasis has been laid on suspected adverse effects on cancer risk and on the signs and symptoms of selenosis. A purported protective effect for cancer and cardiovascular disease was stressed at the turn of the century. However, the lack of evidence of a clear beneficial effect of selenium supplementation did not confirm such a preventive role [7]. In fact, more recent studies have linked selenium overexposure with excess risk of metabolic diseases such as type 2 diabetes [8], hyperlipidemia [9], non-alcoholic fatty liver disease [10] and neurodegenerative disease [5,11]. The possibility that selenium exerts adverse effects on human metabolism, in particular, is supported by converging evidence for type 2 diabetes from both experimental [12] and non-experimental [8] epidemiologic studies. However, the exact amount of selenium exposure that needs to be reached to increase metabolic adverse effects is still partly unclear [6,8]. In addition, evidence suggests the impact of selenium exposure on the thyroid gland, with indications of both adverse and beneficial effects depending on the amount and chemical form of selenium, study population and the thyroid-related endpoint investigated [13,14,15].

In humans, selenium exposure occurs in many different chemical forms [16,17,18,19]. Moreover, because selenium is ubiquitous to the environment, exposure is linked to different sources [5]. This causes it to influence related health effects and underlying biological properties, which are also modified by other dietary constituents [6,7]. However, the main route of exposure for most individuals is diet [20,21]. On the other hand, water, dietary supplements and exposure to environmental sources such as smoking [22] and motorized traffic exhaust [23] may be considered essentially trivial sources [6,20], with the exception of the rare occurrence of occupational exposure [24]. Different biomarkers have been proposed to assess selenium exposure, but the most commonly used and reliable ones are circulating selenium levels in serum, plasma and whole blood [25]. These biomarkers also allow for the assessment of the various selenium chemical species [6]. Other biomarkers that have been adopted to assess selenium contents are nails, toenails and hair [26]. These have several advantages. To begin with, they are informative about long-term selenium exposure. In addition, their sampling and collection are less invasive and, therefore, better tolerated by participants. Nonetheless, they are not suitable for speciation analysis and show a limited correlation with blood selenium levels and dietary selenium intake. For these reasons, they may not be adequate to monitor selenium overexposure [27], possibly owing to their inability to retain some circulating selenium species, such as inorganic ones [28]. Two more suitable indicators have been proposed to assess selenium exposure, i.e., dietary intake and urinary excretion levels. The first can be derived from validated semi-quantitative food frequency questionnaires and may allow us to evaluate selenium intake independently of metabolism and excretion [6]. The second is thought to be an appropriate indicator of recent exposure only [29].

In a group of healthy adults, we investigated the relation between selenium exposure, as assessed through dietary intake and urinary excretion levels, and metabolic endpoints, including blood levels of glucose, total cholesterol, high-density lipoprotein (HDL) cholesterol, low-density lipoprotein (LDL) cholesterol, triglycerides and thyroid-stimulating hormone.

## 2. Materials and Methods

### 2.1. Study Population

We recruited blood donors from the Transfusion Medicine Center ‘Casa del Dono’ of AUSL-IRCCS of Reggio Emilia, Northern Italy, from April 2017 to April 2019. Recruitment followed approval from the Reggio Emilia Ethics Committee (approval no. 2016/0022799) and written informed consent from participants. To be enrolled in the study, all subjects had to be living in the province of Reggio Emilia, be aged 30–60 and be non-smokers. A final number of 148 eligible subjects were accepted to participate in the study. Four participants later withdrew from the study, and an additional seven were excluded because of high urinary cotinine levels (>30 µg/L), inconsistent with the self-declared non-smoker status [30,31]. The final study population was eventually composed of 137 subjects.

After written informed consent was obtained, participants were asked to give a fasting blood and a urine sample. Participants also compiled a detailed questionnaire concerning lifestyle and habits. This included information on height and weight, marital status, education, occupational and residency history, smoking history (never or former smokers) and selenium-containing dietary supplement use. In addition, they were asked to fill in a validated semi-quantitative food frequency questionnaire (European Prospective Investigation into Cancer and Nutrition Food Frequency Questionnaire—EPIC FFQ) already used in previous studies [32,33]. Briefly, the EPIC FFQ allows one to assess dietary habits in the considered population. In particular, it investigates the frequency and amount (selected among three portion sizes) of 188 food items. The related intake of nutrients was calculated using a previously developed ad hoc software. We estimated daily dietary selenium intake by combining the trace-element average in food within the study area and food consumption patterns assessed through the FFQ.

### 2.2. Laboratory Analyses

#### 2.2.1. Analytical Determination of Biochemical Parameters in Blood and Urine

Blood venous and urine samples were collected in a plastic tube and stored at −20 °C until use. Automatized laboratory procedures were used to quantify the following parameters: total cholesterol, HDL-cholesterol, triglycerides, glucose and thyroid-stimulating hormone, while LDL-cholesterol was calculated through the Friedewald formula [34]. Urinary cotinine, a biomarker of tobacco smoking, was measured by Liquid Chromatography with tandem mass spectrometry (LC/MS/MS) (TSQ Quantum Access, Thermo Scientific, Rodano, Italy) [31]. Subjects with mean urinary cotinine ≥ 100 µg/L were classified as active daily smokers. The use of the mean value of urinary cotinine to classify subjects is supported by the relatively long half-life of urinary cotinine (6–22 h) which makes this biomarker quite stable over the day in regular daily smokers.

#### 2.2.2. Analytical Determination of Selenium in Urine

Before analysis, urine samples were thawed at room temperature for 2 h. Each sample was mixed and heated at 37 °C for 30 min to dissolve the sediment. An aliquot of 600 µL was transferred into a 10 mL polyethylene tube and added to 2.4 mL of an aqueous solution of nitric acid 0.05% *v*/*v* prepared by diluting ultrapure nitric acid (69% TraceSelect, Fluka, France), containing 7.5 µg/L of Scandium-45 (45Sc), Yttrium-89 (89Y) and Indium-111 (111In) as internal standards (Inorganic Ventures, Inc., Lakewood, NJ, USA). All solutions were prepared using Milli-Q^®^ ultrapure water (conductivity 0.056 µS/cm) (Merck, Darmstadt, Germany). The urine samples were analyzed by inductively coupled plasma mass spectrometry (ICP-MS) X Series II (Thermo Electron Corporation, Rodano, Italy). The instrument was operated in collision cell mode (CCT-Ked), with 3.7 mL/min of helium used to reduce interference. For each sample, three replicates were run. The calibration curve was in the range of 0.2–70 µg/L. The calibration solutions were obtained by diluting a selenous acid standard solution containing selenium at 1 mg/mL (BDH, VWR International, Milano) with an aqueous solution of nitric acid 0.05% *v*/*v* in the presence of internal standards. The calibration curve was linear with a correlation coefficient ≥0.999. The limits of quantification (LOQs), calculated as ten times the standard deviation of the blank, amounted to 1.2 µg/L. Internal quality assurance was performed using two quality controls (QCs) for metals in urine: Lyphocheck Urine Metals Control, Level-1 (Bio-Rad Laboratories, Anaheim, CA, USA), and Seronorm^®^ Level-1 (Sero AS, Billingstad, Norway). Before analysis, QCs were reconstituted in accordance with manufacturers’ instructions. QC accuracy was between 90% and 110% and precision ranged between 7% and 11%.

### 2.3. Data Analysis

We reported the percentile distribution of urinary selenium concentrations and dietary selenium intake, as well as fasting hematological parameters and urinary cotinine levels. Concerning the occurrence of extreme values, we found a subject with very high triglycerides, 574 mg/dL, and one with very high thyroid-stimulating hormone levels, 15.64 mU/mL, both of which we considered outliers and therefore winsorized at the 99th percentile. Only these two values were changed.

We assessed the association between urinary and dietary selenium concentrations, and between these selenium status indicators and the blood metabolic endpoints of glycemia and HDL-, LDL- and total cholesterol as well as triglycerides, by using crude and multivariable linear regression analyses. For the latter, age, sex, body mass index (BMI—as a continuous variable), cotinine levels and alcohol intake were included in the model as potential confounders. We used linear regression fitted on a restricted cubic spline model and based on three knots at fixed percentiles (10th, 50th and 90th). Moreover, we assessed the statistical precision of the estimates by computing their 95% confidence interval (CI). We used the ‘mkspline’, ‘regress’, ‘xbcrsplinei’ and ‘winsor’ routines of the Stata 17.1 software (Stata Corp., College Station, TX 2021, USA).

## 3. Results

This study included 137 participants, 62 men and 75 women, aged 30–60. Table 1 summarizes the main characteristics of the study population, i.e., sex, age, BMI, smoking habits, consumption of selenium-containing supplements, marital status, educational attainment levels and occupational group, according to the international standard classification of occupations (ISCO). The study population was mainly composed of men and women aged less than 50 years, who were normal weight and had never smoked in their lifetime.

Table 2 shows the distribution of the blood parameters in the study participants and the urinary and dietary selenium concentrations. Median urinary selenium excretion was 22.02 µg/L (interquartile range (IQR) 14.64–37.15 µg/L), while the median daily dietary intake was 78.74 µg (IQR 62.62–101.48 µg/day), with higher values in men compared with women. Men also exhibited higher blood glucose and higher triglyceride levels, along with higher levels of thyroid-stimulating hormone. Conversely, they had lower levels of LDL-, HDL- and total cholesterol.

**Figure 1:**
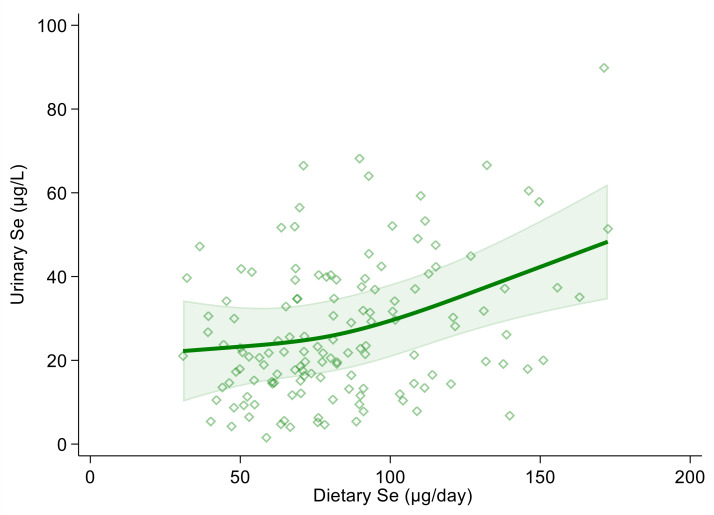
Spline regression analysis of urinary and dietary selenium (Se) levels. Solid line represents multivariable analysis (adjusted for age, sex, body mass index, cotinine levels and alcohol intake) and shaded area indicates upper and lower confidence interval limits.

Using urinary selenium as a dependent variable and dietary selenium intake as an independent one, the linear regression estimate (β coefficient) was 0.19 (95% CI 0.10, 0.27) and 0.18 (95% CI 0.10, 0.27) in the crude and multivariable adjusted models, respectively. Both in the crude and multivariable spline regression analysis, which was adjusted for sex, age, BMI, cotinine levels and alcohol intake, we found a positive and almost linear correlation between urinary and dietary selenium. However, the strength of the association tended to increase at around 100 µg of daily selenium intake and above (Figure 1 and Figure S1).

**Figure 2:**
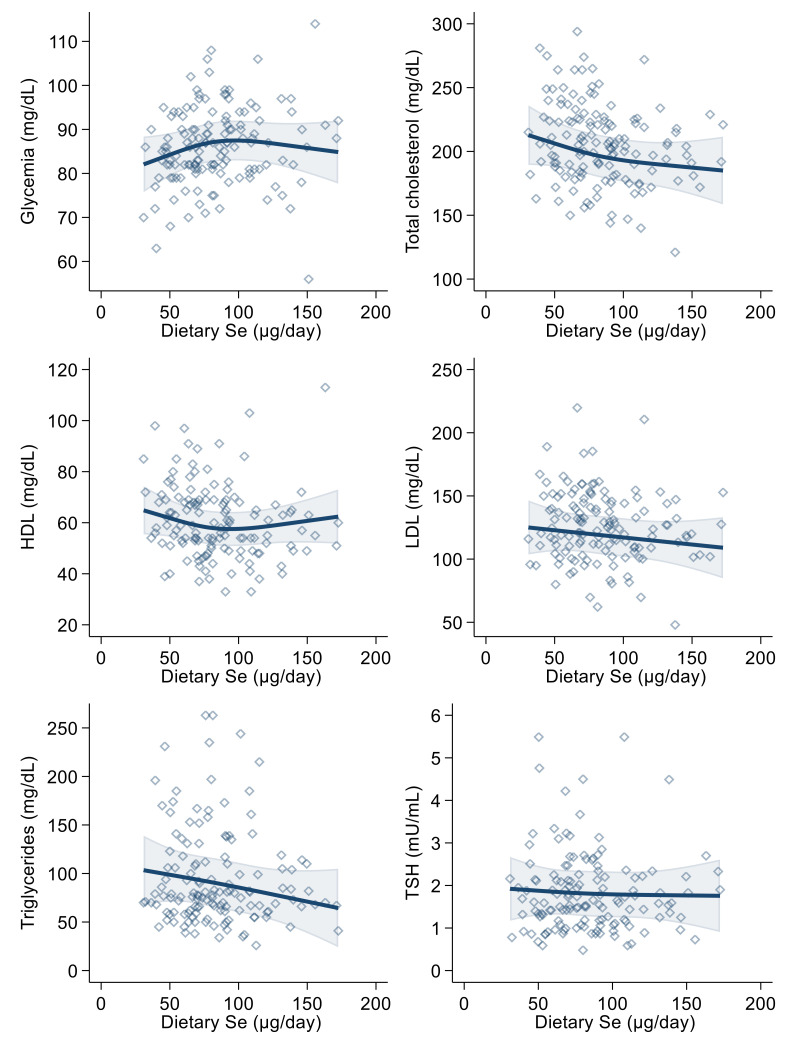
Spline regression analysis of urinary selenium (Se) levels, glycemic and lipid profile variables and thyroid-stimulating hormone (TSH). Solid lines represent multivariable analysis (adjusted for age, sex, body mass index, cotinine levels and alcohol intake) and the shaded area indicates upper and lower confidence interval limits.

In the linear regression analysis, urinary selenium excretion was positively associated with blood glucose, LDL-cholesterol, triglyceride and (slightly and very imprecisely) thyroid-stimulating hormone levels, in both the crude and the multivariable models. The relation was negative with HDL-cholesterol (Table 3). These results were generally confirmed in multivariable spline regression analyses (Figure 2), with limited evidence of any threshold or non-linear shape of the association.

**Figure 3:**
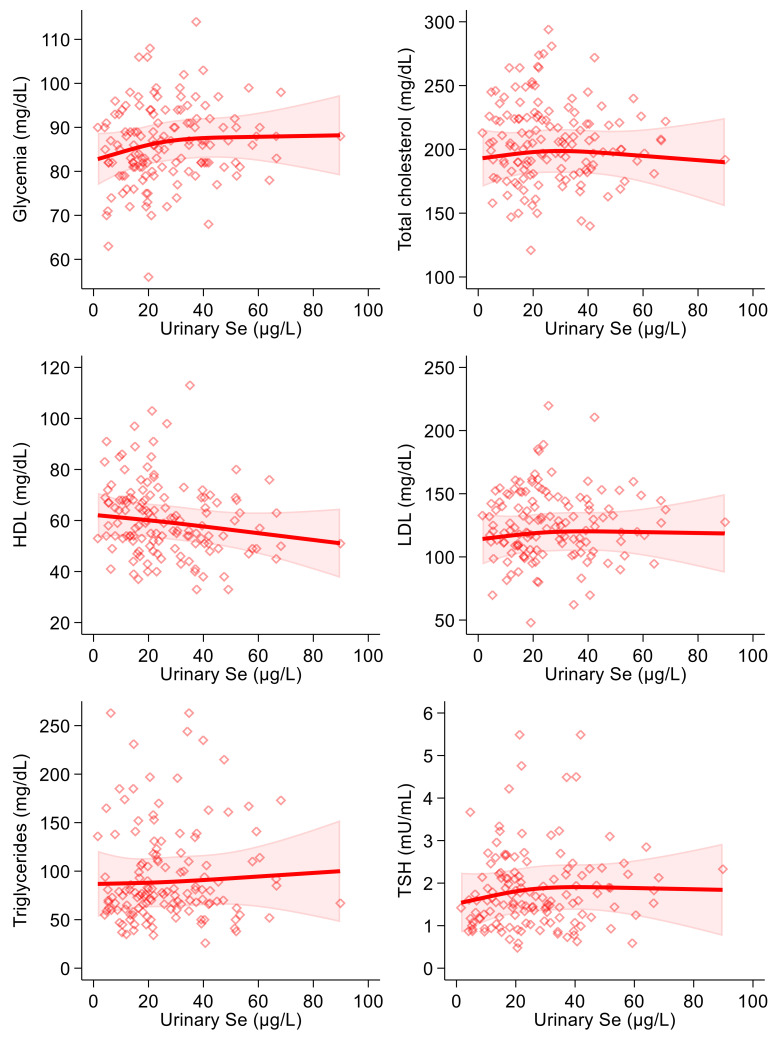
Spline regression analysis of dietary selenium (Se) levels, glycemic and lipid profile variables and thyroid-stimulating hormone (TSH). Solid lines represent multivariable analysis (adjusted for age, sex, body mass index, cotinine levels and alcohol intake) and the shaded area indicates upper and lower confidence interval limits.

As regards the correlation between dietary selenium intake and the endpoints, we found a positive association with glycemia in the linear regression analysis. In the spline regression analysis, furthermore, the shape of the association resembled an inverted U, changing its direction around 90 µg/day (Figure 3). Dietary selenium intake was also slightly inversely correlated with HDL-, LDL- and total cholesterol and with triglycerides in both crude and multivariable analyses (Table 3). In the spline regression analysis, there was an indication of a U-shaped association with blood glucose and of a U-shaped curve with HDL-cholesterol. At the same time, the remaining associations were smoothly negative with the exception of a substantially null association with thyroid-stimulating hormone, with highly scattered intersection points (Figure 3). Concerning thyroid-stimulating hormone, this showed a slightly positive association with urinary selenium in the linear regression analysis, while a negative one was observed with dietary intake. However, all regression estimates for TSH were statistically most unstable, as shown by the wide confidence intervals. In addition, spline regression analysis added limited evidence of a relation of selenium (urinary and particularly dietary) with the blood concentrations of this hormone.

Sex-stratified analyses showed some differences for the male and female population, as reported in Tables S1 and S2. In the linear regression analysis, in men we found a strong positive association between urinary selenium concentration and triglycerides and a weaker but still positive association with glucose, and LDL- and total cholesterol levels. A negative one was observed with HDL-cholesterol. Subgroup analyses in women showed the same positive and negative associations with glucose and HDL-cholesterol levels, respectively, while the association was null with LDL-cholesterol levels, and negative with total cholesterol and triglyceride levels. A negative and very imprecise association was observed with thyroid-stimulating hormone levels. As regards dietary selenium intake, we found slightly positive associations with glucose levels in both men and women, although stronger in the latter, while negative associations emerged with triglycerides as well as HDL-, LDL- and total cholesterol. The association between urinary selenium and thyroid-stimulating hormone levels was found to be slightly negative in men and weakly positive in women, although both estimates were statistically very unstable.

Additionally, the sex-specific spline regression analyses showed differences from the slopes computed for the overall population. Concerning urinary selenium concentration, both men and women experienced the same slightly positive association with blood glucose levels and LDL-cholesterol. Total cholesterol was slightly positively associated with urinary selenium in men, while a negative association was observed in women above 30 µg/L. The association with HDL-cholesterol was different in the two sexes, since we found a U-shaped curve in men, with clear evidence of a decrease up to 30 µg/L and higher values above this apparent threshold. The pattern was almost the opposite in women, with a decreasing trend above a 30 µg/L concentration of urinary selenium. The association with triglycerides was generally positive in men, although a plateau was reached at around 40 µg/L, while in women the relation was negative up to 20 µg/L and then became positive above that value. As in the linear regression analyses, the association between urinary selenium and thyroid-stimulating hormone was different for the two sexes. In fact, an entirely positive and almost linear pattern was observed in men. In women, on the other hand, the positive association flattened around 30 µg/L and the curve then started to decrease, thus showing an inverted U-shaped pattern (Figures S2 and S3).

Furthermore, regarding the associations between dietary selenium intake and health-related endpoints, we found several sex-related differences. Glucose levels were uniformly and positively associated in men, while an inverted U-shaped curve was observed in women above 100 µg/day. The relation with total cholesterol was negative in both subgroups, although its shape was less linear in men. The curve for HDL-cholesterol was similarly U-shaped in men and women, although more flattened in the former subgroup. The association with LDL-cholesterol was U-shaped and inverted U-shaped in men and women, respectively, in both cases changing its direction above 100 µg/day. Triglycerides were negatively related to dietary selenium in both subgroups, although the curves decreased at different intake levels. Concerning the relation with thyroid-stimulating hormone, an almost null association was reported for women, while a negative association was observed in men up to 100 µg/day; above that value, the curve inverted its direction and slightly increased, showing a U-shaped pattern (Figures S4 and S5).

## 4. Discussion

While there is much uncertainty about the role of selenium in human health, convincing evidence has been provided to support its ability to increase the risk of metabolic disease even at unexpectedly low exposure levels. This is clearly the case for type 2 diabetes based on randomized controlled trials [12] and observational studies [8], but it could also be true for hypertension and non-alcoholic fatty liver disease [10,35,36]. An association, either adverse or beneficial, between selenium status and lipid profile is considerably more controversial, due to conflicting findings from both experimental and non-experimental human studies [37,38,39,40,41,42]. In addition, there is still considerable uncertainty about what threshold of selenium exposure can trigger such adverse metabolic effects. Overall, these issues bear on the identification of a safe range of selenium intake, which has generated considerable interest and led to different approaches and standards across countries and the scientific community [6,43,44,45,46].

In this study, evidence appears to substantially validate the assessment of selenium intake through questionnaires on urinary excretion of the element, although such relation has not always been found [24,25,47,48]. It should also be noted that this relation was evident despite the non-irrelevant number of study participants reporting occasional use of selenium-containing dietary supplements. This tends to reduce the reliability of assessments of selenium intake based only on a food frequency questionnaire, as in this case.

Given the positive association between urinary and dietary selenium we detected, it appears surprising that most relations between exposure and health endpoints observed through multivariable regression analysis highlighted an inconsistency between these two indicators of selenium exposure in terms of the direction, strength and statistical precision of the associations. At the same time, it must be noted that the correlation between these two indicators of selenium exposure was positive, but not very strong. The reasons for such partial lack of association could be twofold: first, the different exposure timeframe implied by the two methods, which is longer for dietary selenium intake (approximately one year); secondly, the uncertainty inherent in the dietary assessment methodologies, i.e., an inaccurate evaluation of both participants’ food intake and the selenium content of food items. In addition, such a discrepancy, as well as a lower capacity to correlate with the health endpoints of estimated dietary selenium intake compared with the biomarker, could be due to differences across study participants at two levels: first of all, in terms of diet composition, due to other dietary constituents influencing selenium absorption; secondly, in gastrointestinal absorption capacity. In this study population, therefore, we assume that the biomarker, i.e., urinary selenium concentrations, provides a better estimate of the bioavailable selenium pool in the study participants.

Evidence suggests a detrimental effect of selenium exposure on glucose and lipid metabolism. This is supported by two elements. The first is the positive association between urinary selenium concentrations and both blood glucose and two lipid parameters, such as LDL-cholesterol and triglycerides. The second is the negative association with HDL-cholesterol in both crude and adjusted analyses. This was the case despite the overall amount of selenium exposure falling in the expected range for both the Italian and more generally the European populations [21,26,49], and despite the fact that these associations were weak and generally statistically imprecise. The possibility that selenium may increase glycemia has been suggested by some prospective [50,51] and cross-sectional or case-control studies [50,52,53,54], although not all cohort studies are consistent [6,55]. In addition, a positive association between selenium exposure and glycemia is strongly supported by experimental and non-experimental studies on selenium and type 2 diabetes, with randomized controlled trials consistently showing a relation between selenium supplementation and disease risk [12]. The association was further supported by observational studies providing evidence of increasing diabetes risk above a threshold of selenium exposure at around 80 µg of daily intake [8].

The association of adverse lipid profile endpoints with urinary selenium is not entirely surprising, given the dose–response association between higher blood selenium concentrations and metabolic syndrome, higher triglycerides and LDL-cholesterol, as well as the lower HDL-cholesterol consistently found in the National Health and Nutrition Examination Surveys (NHANES) and other studies [39,52,54,56,57,58]. Conversely, randomized controlled trials have shown little if any effect of selenium supplementation on the lipid profile [38,40]. In those studies, however, high-selenium yeast was used to increase selenium intake, and the specific selenium species released by that source may be different from those characterizing the usual diet. It must also be noted that the other indicator of selenium exposure in our study, namely the assessment of the dietary intake of the element, showed neither a clear nor a consistent association with the lipid profiles. The only exception to this was the inverse association with HDL-cholesterol, which was also the lipid variable most strongly associated with urinary selenium concentrations. Overall, the inverse and almost linear association between urinary selenium exposure and HDL-cholesterol, as well as the positive association with triglyceride levels, can be considered the most relevant and consistent finding of our study with reference to the lipid profile. Moreover, it suggests the deleterious effect of selenium even at the rather low amounts of exposure characterizing the study population, which has so far been considered to be safe according to current recommended dietary values [49,59].

To the metabolic endpoints we also added an endocrine parameter, thyroid-stimulating hormone, whose association with selenium was conflicted across the two exposure indicators used in the study and is substantially unclear. The interaction between selenium intake and thyroid hormone status appears to be complex and largely depends on the amount of exposure, the chemical forms of selenium involved, the specific thyroid hormones under investigation and possible abnormalities in thyroid gland function and disease [6,46]. Selenium administered as selenized yeast (therefore presumably as selenomethionine) decreased thyroid-stimulating hormone levels compared with placebo in a randomized controlled trial [60] and in a subgroup of pregnant women with mild to moderate iodine deficiency [61]. On the other hand, little evidence of any effect emerged from another trial in the UK [19] and a small trial in the US [62]. It must be noted that an inhibitory effect of selenium administration on triiodothyronine and thyroxine and a rise in thyroid-stimulating hormone have been noted in some human studies. However, the exact amount of selenium exposure at which this may occur is unclear [15]. Unfortunately, we did not assess the full spectrum of thyroid hormones in this study. Therefore, we could not test the possibility that the slightly positive and statistically imprecise association between urinary selenium and thyroid-stimulating hormone may be due to a slight inhibitory effect of selenium on triiodothyronine and thyroxine synthesis.

In our study, we also assessed the sex-related differences between selenium and the metabolic endpoints, finding some evidence of a stronger and more adverse association between selenium and metabolic endpoints in men compared with women. In recent years, new evidence has emerged in relation to sexual dimorphism in glucose and lipid metabolism [63,64]. As their molecular mechanisms start to be revealed, some animal and laboratory studies suggest that the tissue distribution of selenium may be affected by hormone levels, and that interactions exist between sex hormones, particularly estrogens, and selenium metabolism and activity parameters [65,66,67,68,69]. This supports the possibility that the effects of selenium differ according to sex, which should be noted with reference to its capacity to alter metabolic disease risk [70,71]. Both in the present study and in previous reports, however, the effect estimates were too statistically unstable to allow for a meaningful assessment of sex as an effect modifier of the adverse metabolic effects of selenium and selenium species.

Among the limitations of this study is its non-experimental design. This precludes the possibility of selectively attributing the trends and changes in health endpoints to variation in selenium exposure, given the potential for confounding due to unmeasured dietary and lifestyle factors. On the other hand, the presence of clinical and subclinical disease biasing both selenium intake and metabolism is very unlikely to have occurred in a substantial way in our study population. In fact, our carefully selected ‘healthy’ volunteers were also closely monitored with reference to their blood donation. Another limitation is that in this report we did not consider the chemical species of selenium found in foods and in the body (blood and urine). This should be assessed in the context of the limited information about selenium speciation in food, and bearing in mind that the determination of the chemical form of the element in urine is very rarely performed. The specificity of both nutritional and toxic properties of selenium critically depends on their chemical species [16,17,72,73], and this may also be true for the adverse effects on metabolic parameters. Finally, the potential mechanisms underpinning the adverse metabolic effects of selenium should be investigated in a more in-depth fashion, focusing on the possible influence of selenium species on redox status [74,75,76], protein integrity [77] and the microbiome—a less investigated but potentially interesting lead due to its connection with metabolic and chronic diseases [78,79].

## 5. Conclusions

In conclusion, our findings provide evidence for a positive and almost linear associations between urinary selenium and glycemic and triglyceride levels, and a negative relation with LDL-cholesterol concentrations. On the other hand, dietary selenium intake was more weakly and non-linearly associated with most of the endpoints considered. If confirmed by further studies, our results may add to the available evidence at two levels: first of all in reconsidering the safer levels of selenium intake, and secondly in establishing which indicators of selenium exposure are more suitable for the assessment of its toxic effects.

## Figures and Tables

**Table 1:** Characteristics of the study population and mean urinary and dietary selenium (Se) concentrations for each subgroup.

Characteristics	All				Men				Women			
	N	%	Urinary Se (µg/L)	Dietary Se (µg/day)	N	%	Urinary Se (µg/L)	Dietary Se (µg/day)	N	%	Urinary Se (µg/L)	Dietary Se (µg/day)
Overall	137	100	26.77	84.09	62	45.3	29.0	89.97	75	54.7	24.92	79.23
Age												
<50 years	80	58.4	27.23	86.13	39	62.9	30.17	90.94	41	54.7	24.44	81.55
≥50 years	57	41.6	26.12	81.24	23	37.1	27.04	88.32	34	45.3	25.50	76.44
BMI												
<25 kg/m^2^	74	54.0	25.59	82.21	32	51.6	28.47	91.10	42	56.0	23.39	75.44
≥25 kg/m²–<30 kg/m^2^	50	36.5	28.58	84.22	27	43.6	29.49	87.04	23	30.7	27.52	80.92
≥30 kg/m^2^	13	9.5	26.52	94.31	3	4.8	30.40	104.27	10	13.3	25.36	91.32
Smoking habits												
Never	101	73.7	26.10	83.95	45	72.6	28.77	88.60	56	74.7	23.95	80.21
Former	36	26.3	28.66	84.50	17	27.4	29.64	93.60	19	25.3	27.79	76.36
Selenium supplement users												
No	94	68.6	25.06	87.56	46	74.2	29.80	94.65	48	64.0	22.46	80.76
Yes	23	16.8	29.08	80.14	6	9.7	27.75	72.37	17	22.7	29.55	82.88
Former	20	14.6	27.48	72.34	10	16.1	26.11	78.97	10	13.3	28.85	65.71
Marital status												
Married/unmarried partner	97	70.8	26.78	83.10	44	71.0	29.72	87.69	53	70.7	24.34	79.28
Single	26	19.0	27.65	87.72	12	19.4	28.80	104.28	14	18.7	26.66	73.52
Separated/divorced	14	10.2	25.07	84.26	6	9.6	24.20	78.05	8	10.7	25.73	88.91
Educational level												
Elementary school	2	1.5	37.26	146.96	2	3.2	37.26	146.96	-	-	-	-
Middle school	20	14.6	25.99	84.79	8	12.9	29.46	80.08	12	16.0	23.67	87.92
High school	66	48.2	23.87	82.73	28	45.2	24.98	90.92	38	50.7	23.05	76.70
College or more	49	35.8	30.57	83.07	24	38.7	32.87	87.40	25	33.3	28.37	78.92
Occupation (ISCO)												
Managers	9	6.6	21.30	84.83	6	9.7	21.42	80.45	3	4.0	21.04	93.60
Professionals	26	19.0	30.16	91.07	12	19.4	33.81	104.05	14	18.7	27.03	79.93
Technicians/associate professionals	21	15.3	25.96	82.74	11	17.7	23.41	89.03	10	13.3	28.75	75.82
Clerical support workers	43	31.4	25.78	79.34	12	19.4	32.10	82.18	31	41.3	22.99	78.24
Service and sales workers	11	8.0	26.65	71.98	2	3.2	22.71	81.07	9	12.0	27.52	69.96
Craft and related trade workers	10	7.3	22.16	80.73	8	12.9	24.80	82.40	2	2.7	11.61	74.09
Plant and machine operators	11	8.0	35.63	94.40	8	12.9	36.22	100.51	3	4.0	34.05	78.12
Elementary occupations	6	4.4	21.92	100.44	3	4.8	25.78	85.25	3	4.0	18.07	115.63

Abbreviations: ISCO, International Standard Classification of Occupations.

**Table 2:** Median, 25th and 75th percentile of urinary and dietary selenium distribution and fasting blood parameters in the study population (*n* = 137).

Parameter	25th	Median	75th
*All participants*			
Selenium			
Dietary intake (µg/day)	62.62	78.74	101.48
Urinary concentration (µg/L)	14.64	22.02	37.15
Blood parameter			
Glycemia (mg/dL)	81	86	91
Total cholesterol (mg/dL)	184	204	224
HDL-cholesterol (mg/dL)	51	59	69
LDL-cholesterol (mg/dL)	109	124	144
Triglycerides (mg/dL)	62	78	112
Thyroid-stimulating hormone (mU/mL)	1.18	1.59	2.21
Urinary cotinine levels (µg/L)	0.05	0.27	0.86
*Men*			
Selenium			
Dietary intake (µg/day)	69.77	88.37	108.28
Urinary concentration (µg/L)	16.72	24.21	39.20
Blood parameter			
Glycemia (mg/dL)	82	88	94
Total cholesterol (mg/dL)	177	192	219
HDL-cholesterol (mg/dL)	46	52	58
LDL-cholesterol (mg/dL)	101	120	142
Triglycerides (mg/dL)	67	85	135
Thyroid-stimulating hormone (mU/mL)	1.16	1.75	2.34
Urinary cotinine levels (µg/L)	0.05	0.20	0.77
*Women*			
Selenium			
Dietary intake (µg/day)	54.77	71.06	91.68
Urinary concentration (µg/L)	13.30	21.30	34.66
Blood parameter			
Glycemia (mg/dL)	79	85	89
Total cholesterol (mg/dL)	192	210	227
HDL-cholesterol (mg/dL)	57	67	73
LDL-cholesterol (mg/dL)	112	125	146
Triglycerides (mg/dL)	58	73	106
Thyroid-stimulating hormone (mU/mL)	1.18	1.54	2.16
Urinary cotinine levels (µg/L)	0.05	0.32	0.94

**Table 3:** Linear regression analysis of glycemia, lipid profile variables and thyroid-stimulating hormone versus urinary selenium (Se) concentration and dietary Se intake biomarkers as independent variables. Crude model and adjusted for age, sex, body mass index (BMI), cotinine levels and alcohol intake along with their 95% confidence interval (CI).

Linear Regression Analysis		Crude		Adjusted
Urinary Se concentration (µg/L)	β	(95% CI)	β	(95% CI)
Glycemia (mg/dL)	0.08	(−0.01, 0.18)	0.08	(−0.02, 0.16)
Total cholesterol (mg/dL)	−0.10	(−0.43, 0.22)	−0.01	(−0.32, 0.32)
HDL-cholesterol (mg/dL)	−0.18	(−0.33, −0.04)	−0.12	(−0.25, 0.003)
LDL-cholesterol (mg/dL)	0.03	(−0.25, 0.32)	0.07	(−0.21, 0.36)
Triglycerides (mg/dL) *	0.16	(−0.35, 0.67)	0.14	(−0.35, 0.62)
Thyroid-stimulating hormone (mU/mL) *	0.005	(−0.005, 0.015)	0.005	(−0.005, 0.015)
Dietary Se intake (µg/day)	β	(95% CI)	β	(95% CI)
Glycemia (mg/dL)	0.04	(−0.01, 0.09)	0.02	(−0.02, 0.07)
Total cholesterol (mg/dL)	−0.22	(−0.39, −0.05)	−0.20	(−0.37, −0.03)
HDL-cholesterol (mg/dL)	−0.07	(−0.15, 0.01)	−0.02	(−0.09, 0.05)
LDL-cholesterol (mg/dL)	−0.11	(−0.26, 0.04)	−0.11	(−0.27, 0.04)
Triglycerides (mg/dL) *	−0.16	(−0.43, 0.11)	−0.27	(−0.53, −0.02)
Thyroid-stimulating hormone (mU/mL) *	−0.001	(−0.007, 0.004)	−0.001	(−0.007, 0.004)

* Linear regression estimates calculated using winsorized values.

## Data Availability

The data presented in this study may be available on reasonable request from the corresponding author. The data are not publicly available due to privacy and legal restrictions.

## Supplementary Materials

The following are available online at https://www.mdpi.com/article/10.3390/antiox10081193/s1.
