# Supplementary material for: Associations between Urinary and Dietary Selenium and Blood Metabolic Parameters in a Healthy Northern Italy Population
Source: Antioxidants (Basel). 2021 Jul 26;10(8):1193. doi: 10.3390/antiox10081193 (PMC8389012; doi:10.3390/antiox10081193)

Supplemental Material

Table S1. Linear regression analysis of glycaemia, lipid profile variables and TSH versus urinary selenium (Se) excretion and dietary selenium biomarkers as independent variables for the male population (n=62). Crude model and adjusted for age, body mass index (BMI), cotinine levels, and alcohol intake along with their 95% confidence interval (CI).

	**Crude**		**Adjusted**	
**Urinary Se concentration (µg/L)**	**β**	**95% CI**	**β**	**95% CI**
Glycemia (mg/dL)	0.05	(-0.10, 0.19)	0.07	(-0.06, 0.19)
Total cholesterol (mg/dL)	0.12	(-0.38, 0.62)	0.21	(-0.30, 0.71)
HDL-cholesterol (mg/dL)	-0.10	(-0.26, 0.07)	-0.09	(-0.25, 0.06)
LDL-cholesterol (mg/dL)	0.13	(-0.35, 0.60)	0.17	(-0.32, 0.66)
Triglycerides (mg/dL)	0.38	(-0.49, 1.24)	0.53	(-0.34, 1.39)
Thyroid-stimulating hormone (mU/mL)	0.01	(-0.001, 0.03)	0.01	(-0.01, 0.03)
**Dietary Se intake (µg/day)**	**β**	**95% CI**	**β**	**95% CI**
Glycemia (mg/dL)	0.02	(-0.07, 0.10)	0.01	(-0.06, 0.08)
Total cholesterol (mg/dL)	-0.21	(-0.50, 0.07)	-0.22	(-0.51, 0.06)
HDL-cholesterol (mg/dL)	-0.01	(-0.11, 0.08)	-0.02	(-0.10, 0.07)
LDL-cholesterol (mg/dL)	-0.14	(-0.42, 0.13)	-0.15	(-0.43, 0.13)
Triglycerides (mg/dL)	-0.25	(-0.75, 0.25)	-0.27	(-0.77, 0.23)
Thyroid-stimulating hormone (mU/mL)	-0.004	(-0.01, 0.004)	-0.004	(-0.01, 0.004)

Table S2. Linear regression analysis of glycaemia, lipid profile variables and TSH versus urinary selenium (Se) excretion and dietary selenium biomarkers as independent variables for the female population (n=75). Crude model and adjusted for age, body mass index (BMI), cotinine levels, and alcohol intake along with their 95% confidence interval (CI).

	**Crude**		**Adjusted**	
**Urinary Se concentration (µg/L)**	**β**	**95% CI**	**β**	**95% CI**
Glycemia (mg/dL)	0.09	(-0.02, 0.21)	0.09	(-0.03, 0.21)
Total cholesterol (mg/dL)	-0.23	(-0.66, 0.20)	-0.23	(-0.66, 0.21)
HDL-cholesterol (mg/dL)	-0.17	(-0.37, 0.04)	-0.16	(-0.36, 0.04)
LDL-cholesterol (mg/dL)	-0.04	(-0.40, 0.31)	-0.03	(-0.39, 0.33)
Triglycerides (mg/dL)	-0.24	(-0.80, 0.33)	-0.24	(-0.80, 0.33)
Thyroid-stimulating hormone (mU/mL)	-0.003	(-0.02, 0.01)	-0.0002	(-0.01, 0.01)
**Dietary Se intake (µg/day)**	**β**	**95% CI**	**β**	**95% CI**
Glycemia (mg/dL)	0.05	(-0.01, 0.10)	0.04	(-0.02, 0.10)
Total cholesterol (mg/dL)	-0.18	(-0.39, 0.03)	-0.17	(-0.39, 0.04)
HDL-cholesterol (mg/dL)	-0.04	(-0.15, 0.06)	-0.02	(-0.12, 0.08)
LDL-cholesterol (mg/dL)	-0.08	(-0.26, 0.09)	-0.07	(-0.25, 0.10)
Triglycerides (mg/dL)	-0.21	(-0.51, 0.08)	-0.30	(-0.57, -0.03)
Thyroid-stimulating hormone (mU/mL)	0.001	(-0.006, 0.007)	0.001	(-0.006, 0.008)

Abbreviations: HDL, high-density lipoprotein; LDL, low-density lipoprotein**;** TSH, thyroid-stimulating hormone

Figure S1. Spline regression analysis of urinary and dietary Se levels. Solid line represents crude analysis with upper and lower confidence interval limits.

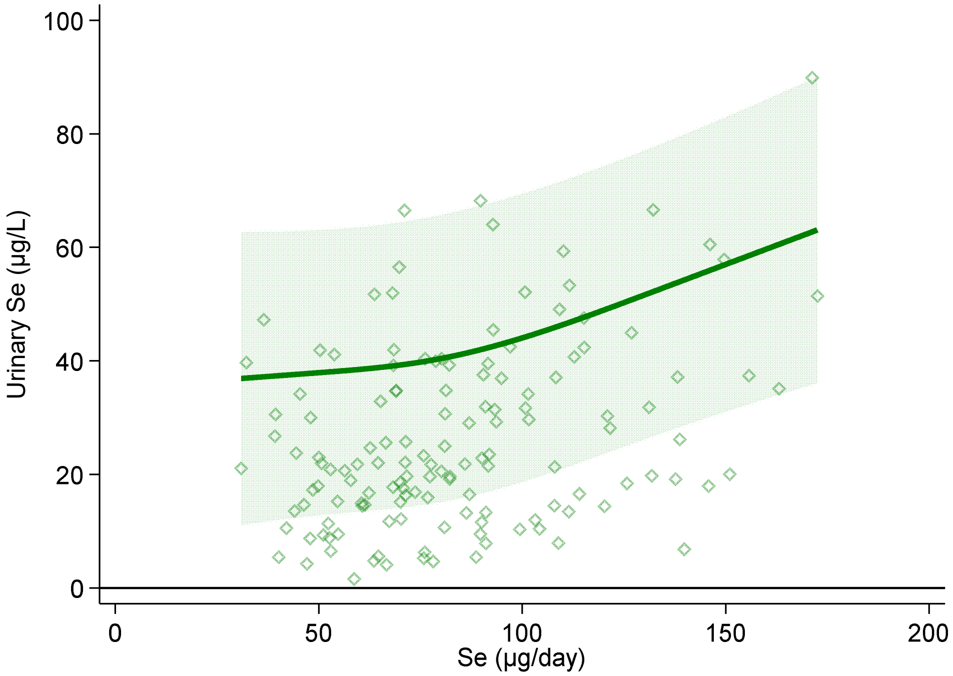

Figure S2. Spline regression analysis of urinary Se levels and glycemic, lipid profile variables and thyroid-stimulating hormone (TSH) in males (n=62). Solid lines represent multivariable analysis (adjusted for age, body mass index, cotinine levels, and alcohol intake) with upper and lower confidence interval limits.

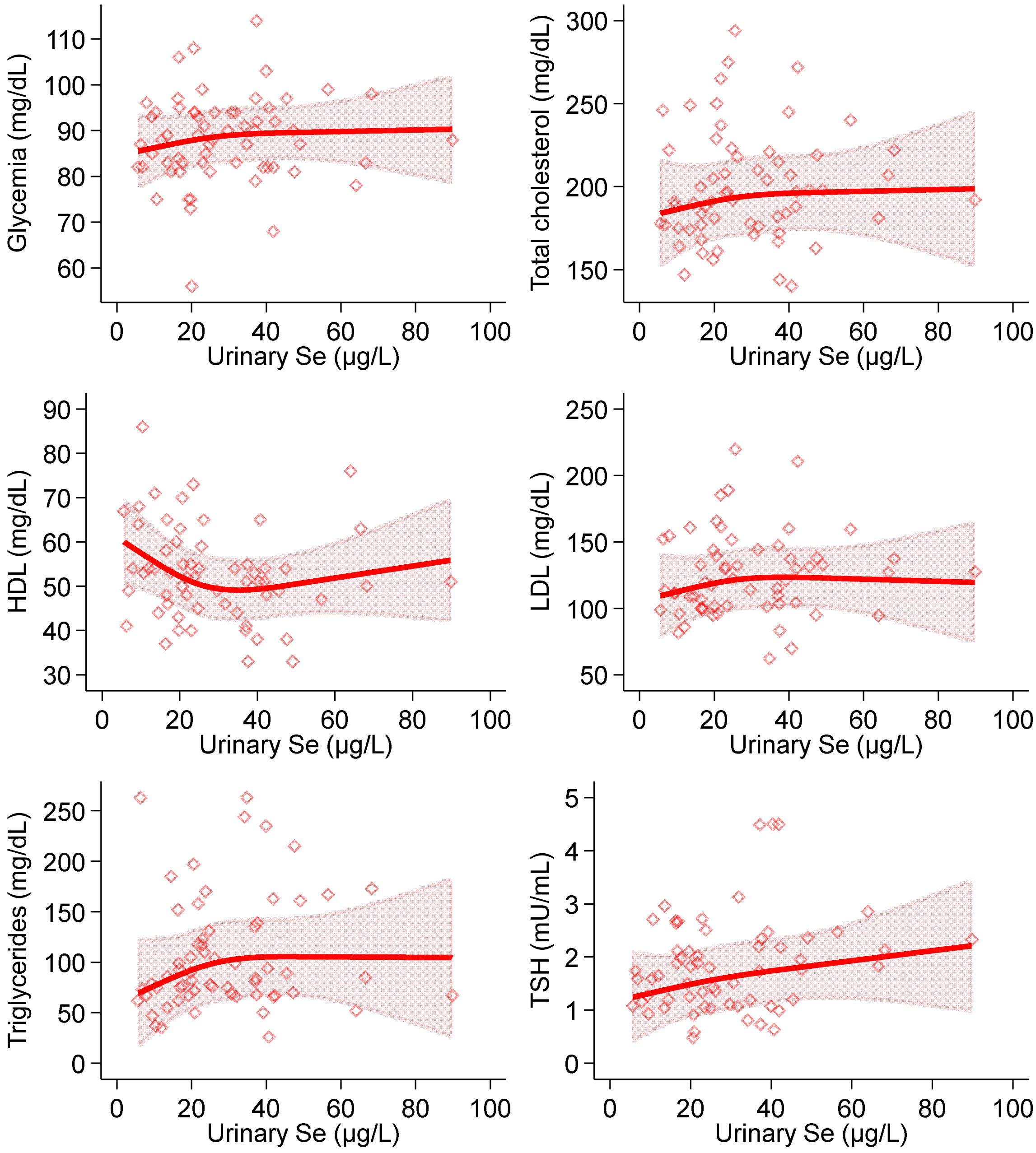

Figure S3. Spline regression analysis of urinary Se levels and glycemic, lipid profile variables and thyroid-stimulating hormone (TSH) in females (n=75). Solid lines represent multivariable analysis (adjusted for age, body mass index, cotinine levels, and alcohol intake) with upper and lower confidence interval limits.

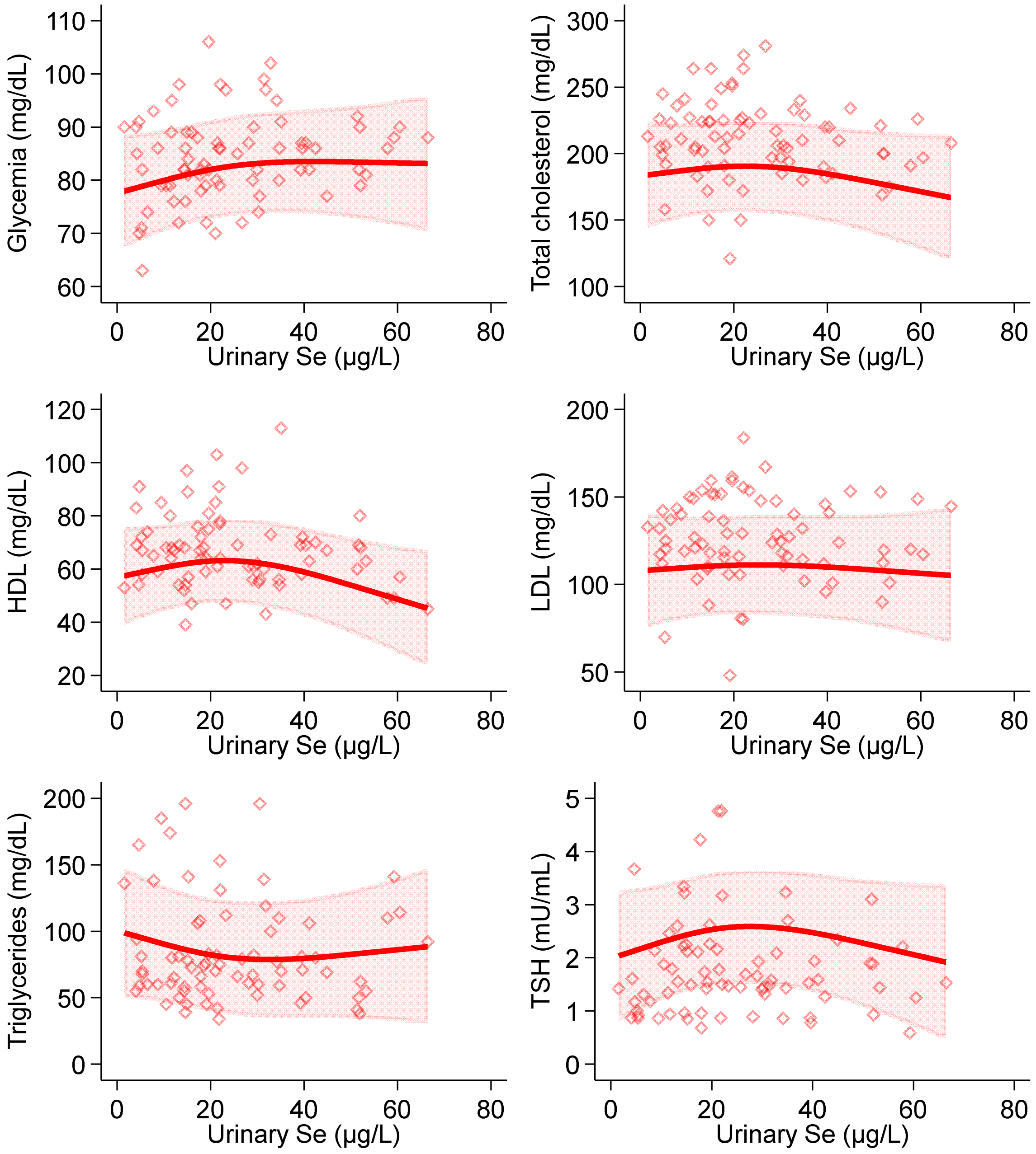

Figure S4. Spline regression analysis of dietary Se levels and glycemic, lipid profile variables and thyroid-stimulating hormone (TSH) in males (n=62). Solid lines represent multivariable analysis (adjusted for age, body mass index, cotinine levels, and alcohol intake) with upper and lower confidence interval limits.

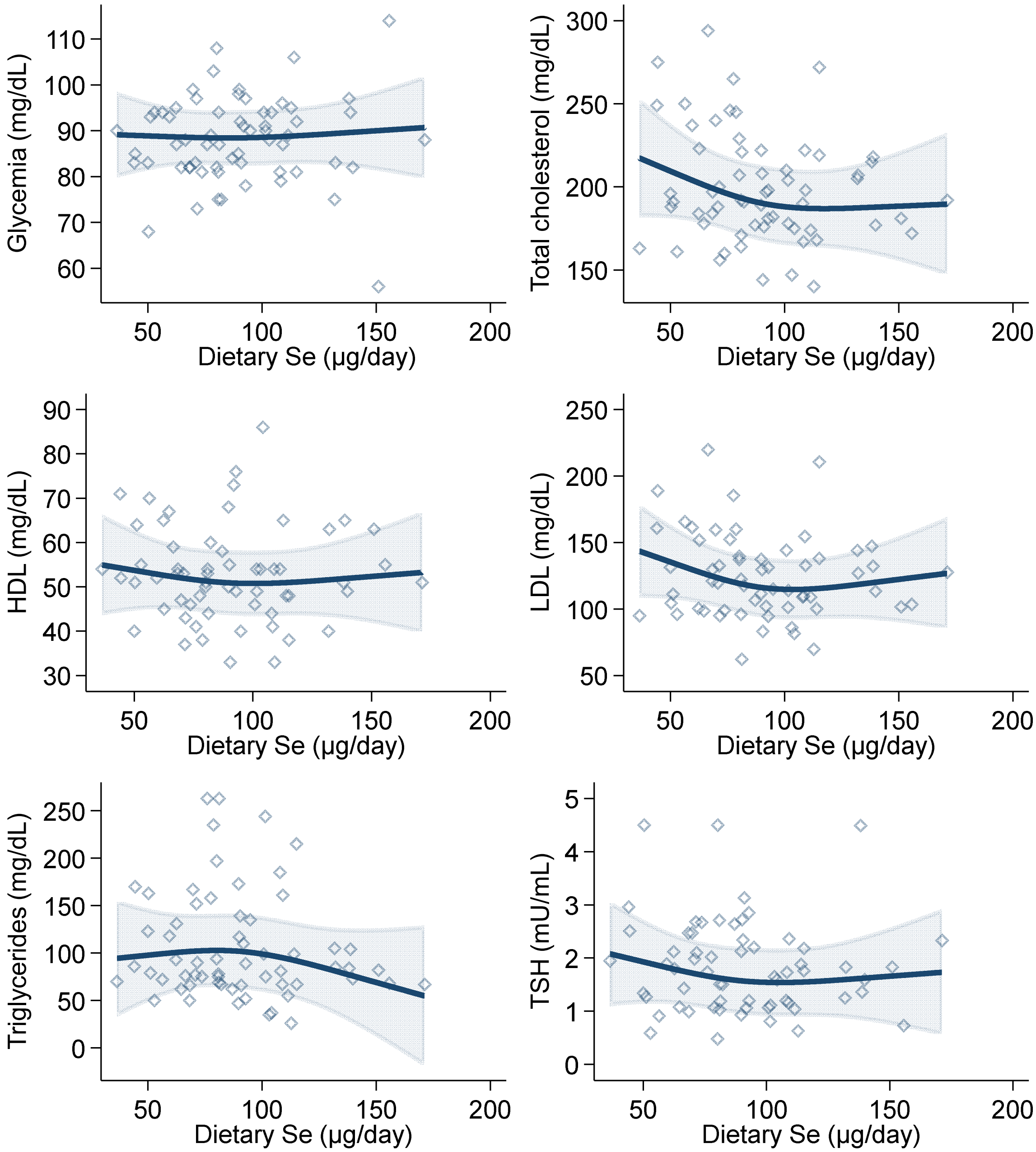

Figure S5. Spline regression analysis of dietary Se levels and glycemic, lipid profile variables and thyroid-stimulating hormone (TSH) in females (n=75). Solid lines represent multivariable analysis (adjusted for age, body mass index, cotinine levels, and alcohol intake) with upper and lower confidence interval limits.

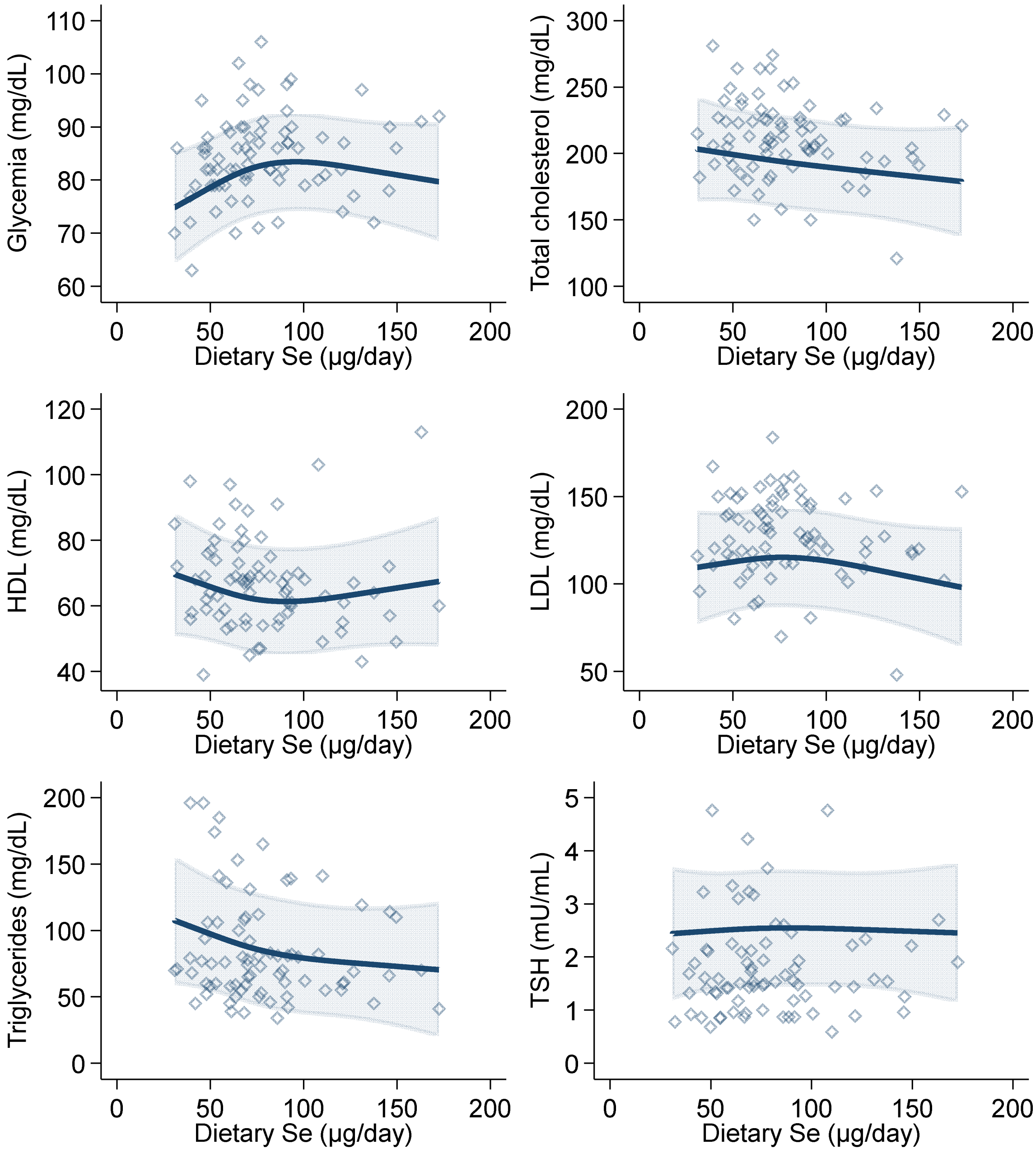

Supplement: Supplementary file 1 [file antioxidants-10-01193-s001.zip › antioxidants-1309130-supplementary.pdf]
